# Synthesis and Phytogrowth Properties of Oxabicyclic Analogues Related to Helminthosporin

**DOI:** 10.3390/molecules14010160

**Published:** 2009-01-01

**Authors:** Luiz Cláudio Almeida Barbosa, Leonardo Brandão Nogueira, Célia Regina Álvares Maltha, Róbson Ricardo Teixeira, Antônio Alberto Silva

**Affiliations:** 1 Department of Chemistry, Federal University of Viçosa, 36570-000, Viçosa, Minas Gerais, Brazil; E-mail: leonardolbn@yahoo.com.br (L-B. N.); teixeir5@yahoo.com.br (R-R. T.); crmaltha@ufv.br (C-A. M.); 2 Plant Science Department, Federal University of Viçosa, 36570-000, Viçosa, Minas Gerais, Brazil;E-mail: aasilva@ufv.br (A-A. S.)

**Keywords:** Helminthosporic acid, Helminthosporol, Helminthosporal, Helminthosporins oxabicycle, Herbicide, [4+3] Cycloaddition, Oxyallyl cation, Phytogrowth properties.

## Abstract

This investigation describes the synthesis and biological evaluation of a series of oxabicyclic analogues related to the helminthosporins. Four oxabicycles were prepared by [4+3] cycloaddition of an oxyallyl carbocation, generated *in situ* from 2,4-dibromopentan-3-one, with selected furans. Functional group manipulations of the oxabicyclic architecture generated nine further derivatives. The phytotoxic properties of these oxabicycles were evaluated as their ability to interfere with the growth of *Sorghum bicolor* and *Cucumis sativus* seedlings. In both species, the most active compounds were oxabicycles possessing a carbonyl group conjugated with a double bond.

## Introduction

Microorganisms are capable of producing a huge variety of structurally diverse secondary metabolites as recently described in an excellent review by Strange [1]. These metabolites have been considered as potential compounds for use as herbicides or as novel lead structures for the development of weed controllers [1,2,3].

**Figure 1:**
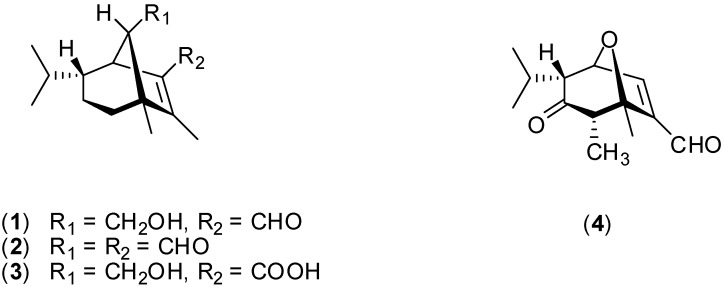
Structures of compounds **1-4** mentioned in the text.

Chemical investigations on the cultured broth of the plant pathogenic fungus *Cochliobolus sativus* (Ito & Kurib.) Drechsler ex Dastur (anamorph: *Bipolaris sorokiniana (*Sacc.) Shoem.) led to the isolation of both helminthosporal (**1**) [4] and helminthosporol (**2**) [5] (Figure 1). Biological assays conducted with compound **2 **revealed that this metabolite is a plant growth regulator, which promotes the development of rice seedlings at concentrations ranging between 10 ppm and 300 ppm [6]. Gibberellins, which constitute another class of fungal metabolites with potential applications in agriculture, exhibit this type of activity too. For instance, at 50 ppm helminthosporol (**2**) causes root growth inhibition and at 300 ppm it prevents root emergence. Moreover, compound **2** also inhibits shoot growth of wheat seedlings above 30 ppm [6]. Helminthosporal (**1**) is a crop-destroying toxin that inhibits the respiration of roots and coleoptiles of barley and wheat of different strains. The toxin **1 **also inhibits the respiration of root of storage tissue from radish, squash, bean, lettuce, turnip, sweet and white potato [7]. 

It has also been demonstrated that helminthosporal (**1**) and helminthosporic acid (**3**) inhibit the cholesterol acyltransferase (ACAT) activity in rat liver microsoms. However a relationship between this activity and the growth-regulating effects in plants has not yet been established [8]. Further studies demonstrated that several analogues of compound **3** displayed gibberellin-like properties [9, 10]. Studies carried out by Cutler and co-workers suggested that the phytotoxicity of *Cochliobolus sativus* may due, in part, to the hemiacetal called prehelminthosporol, a compound that causes chlorosis and necrosis in both beans and corn [11].

Within the frame of a long-established research of new active principles to control weeds [12,13,14,15,16,17,18,19,20], and taking into consideration the phytotoxic activities associated with compounds **1**-**3 **previously mentioned, we have undertaken the synthesis of several analogues related to the toxins **1-3**. The effect of the synthesized compounds on monocotyledonous and dicotyledonous have been evaluated [21,22,23]. It has been found that some analogues displayed significant phytotoxicity. For instance, compound **4** inhibited root elongation of *Sorghum bicolor* by 82% at 10^-3^ mol L^-1^. 

The encouraging results aforementioned prompted us to further our investigations on the potential phytotoxicity of helminthosporin analogues. In this paper, we describe the synthesis and biological evaluation of oxa-analogues related to toxins **1**-**3**. Selected aspects concerning their structure-activity relationships are also discussed.

## Results and Discussion

### Synthesis

**Scheme 1:**
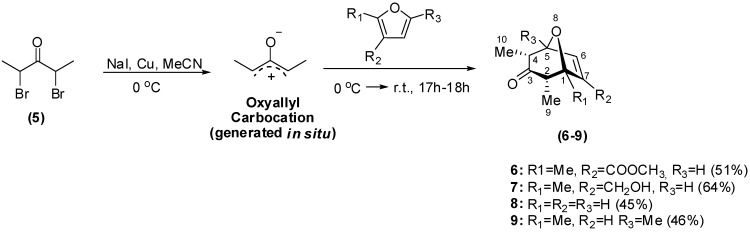
Reaction conditions for the preparation of oxabycicles **6-9**.

The [4+3] cycloaddition between allyl cations and dienes constitutes a powerful methodology to access compounds containing seven-membered rings [24,25,26,27]. This synthetic route has led to the preparation of several biologically active compounds [21,22,23]. Thus, in the present work, the cycloaddition reactions between the oxyallyl carbocation itself, generated from 2,4-dibromopentan-3-one (**5**), and different furans, afforded the oxabicycles **6-9** in variable yields (Scheme 1).

Detailed IR, NMR and MS analyses confirmed the structures of all synthesized oxabicycles. 2D-NMR experiments proved essential for definitively assigning various hydrogen and carbon resonances. The major cycloadducts isolated were *endo* isomers, in which the 2-methyl and the 4-methyl groups occupied equatorial positions. Although the [4+3] cycloaddition reactions can also produce *exo-*cycloadducts in small amounts [25,26,27], they were not isolated or detected in the present study. As previously reported, in the *endo* isomer, the magnitude of the coupling constant involving hydrogen-4 and hydrogen-5 (*J_H4-H5_*) lie between 4.5 to 5.0 Hz (vide Scheme 1 for numbering) [28]. Similar values of *J_H4-H5 _*were found for cycloadducts synthesized in this study (see Experimental Section).

**Scheme 2:**
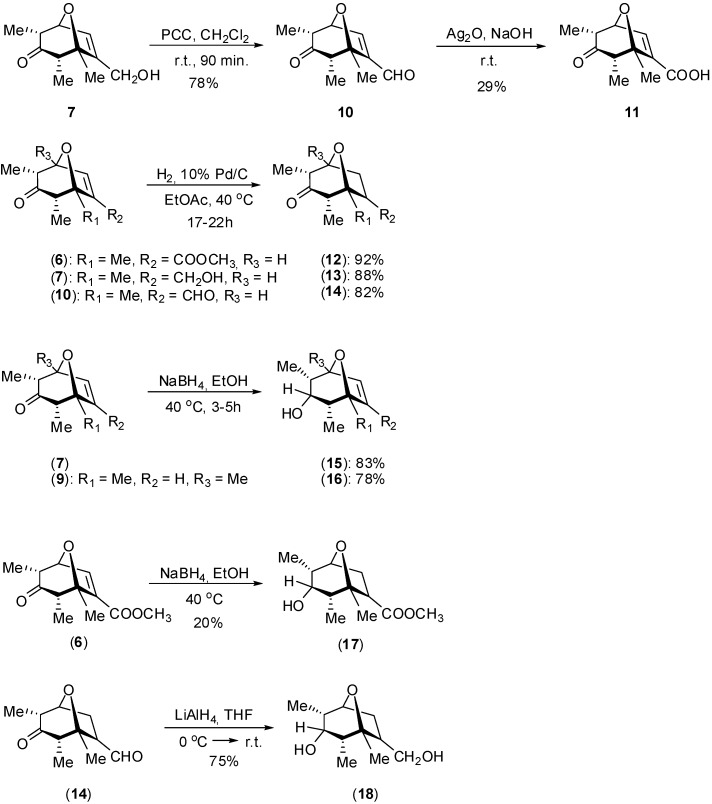
Synthetic functional group interconversions of oxabycicles **6, 7 **and **9**.

Compounds **6, 7 **and **9** were subsequently submitted to straightforward functional group manipulations (Scheme 2) which not only afforded new derivatives, but also allowed us to evaluate the significance of different functionalities for the biological activity.

The oxidation of aldehyde **10** with silver oxide afforded the carboxylic acid **11** in moderate yield. Compound **11** could also be prepared from the hydrolysis of ester **6** albeit with modest 32% yield. The reaction of compound **6** with NaBH_4_ led to the formation of a complex mixture from which the saturated ester **17** could be isolated in 20% yield. In contrast, other compounds shown in Scheme 2 were obtained in good yields. The identity of oxabicycles **10**-**18** was established­ using a combination of NMR, IR and MS analyses. Since the preparation of derivatives **10**-**14**, **17** and **18** has not been previously reported, they are fully detailed in the Experimental section.

### Biological Evaluation of the Synthesized Compounds as Potential Phytogrowth Regulators

The potential phytotoxicity of oxabycicles **6**-**18 **was evaluated as the ability to interefere at 5 x 10^-4^ mol L^-1 ^with the radicle growth of *Cucumis sativus* (a dicotyledonous species) and *Sorghum bicolor* (a monocotyledonous species). The effects of the compounds studied for phytotoxicity on the dicotyledonous species are summarized in Table 1.

**Table 1:** Effect of Oxabicycles **6**-**18** on Radicle Growth of *Cucumis sativus* at 5 x 10^-4^ mol L^-1^

Compounds	radicle length (cm)^*^	inhibition (%)^**^
**6**	2.92 f	37.61
**7**	4.55 abc	2.78
**8**	4.62 abc	1.28
**9**	4.16 abcd	8.89
**10**	2.97 ef	36.54
**11**	3.32 def	29.06
**12**	3.60 cdef	23.08
**13**	3.45 def	26.28
**14**	4.59 abc	1.92
**15**	4.78 ab	-2.14
**16**	3.62 cdef	22.65
**17**	5.05 a	-7.90
**18**	3.95 bcde	15.60
Control	4.62 abc	
Water	4.68 ab	
CV (%)	9.96	

^* ^Means in the same column with the same letter are significantly different at *P =* 0.05% by Tukey’s test. ^**^Values are expressed as percentages differences from the control.

None of the compounds caused any significant effect on the germination rate*.* However, with the exception of compounds **15** and **17**, all the remaining oxabicycles inhibited the radicle growth of the tested species. Considering inhibitory effects at the concentration of 5 x 10^-4^ mol L^-1^, compounds **6** and **10 **were the most effective causing, respectively, 37.61% and 36.54% inhibition. Interestingly, these two compounds share a common structural feature i.e. the presence of a carbonyl group conjugated with a double bond. According to Macías and co-workers [29], an *α,β*-unsaturated carbonyl moiety could act as a Michael acceptor with a putative nucleophilic residue of a biomolecule. Consequently, this moiety may be an important requirement for enhance the desired inhibitory activity.

Based on the study of several helminthosporic acid analogues, Turner and co-workers [10] have proposed that the double bond has only a minor effect on the biological activity. In order to test the significance of the double bond on the inhibitory activity of these oxabicycles derivatives **6**, **7**, and **10** were prepared by hydrogenation. Significantly, removal of unsaturation decreased the biological activities of compounds **6** and **10**, a fact that can be ascribed to the loss of conjugation with the carbonyl group. In contrast, hydrogenation of compound **7** led to a derivative with higher inhibitory activity. One possibility is that increase in conformational flexibility improved the inhibitory activity by inducing a better fit within the target receptor [30]. The phytotoxic activity of oxabycicles **6-18** was further investigated on *Sorghum bicolor*, and results are presented in Table 2. 

**Table 2:** Effect of Oxabicycles **6**-**18** on Radicle Growth of *Sorghum bicolor* at 5 x 10^-4^ mol L^-1^.

Compounds	radicle length (cm)^*^	inhibition (%)**
**6**	3.90 ab	13.33
**7**	3.25 bc	27.78
**8**	3.19 bc	29.11
**9**	3.37 bc	25.11
**10**	2.16 d	52.00
**11**	3.98 ab	11.55
**12**	2.75 cd	38.89
**13**	3.26 bc	27.56
**14**	2.77 cd	38.44
**15**	3.18 bc	29.33
**16**	3.72 ab	17.33
**17**	4.41 a	2.00
**18**	3.21 bc	28.67
Control	4.54 a	
Water	4.50 a	
CV (%)	10.70	

^* ^Means in the same column with the same letter are not significantly different at *P =* 0.05% by Tukey’s test. ^ **^Values are expressed as percentages different from the control.

**Figure 2:**
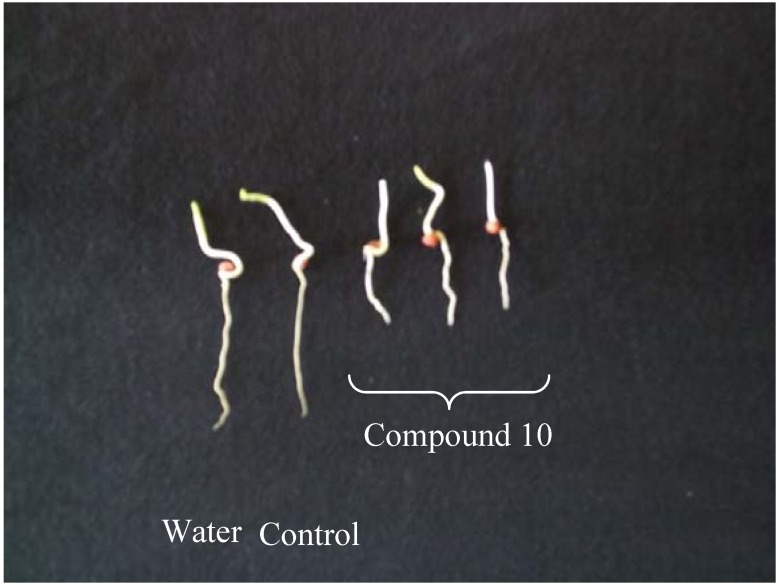
Effect of aldehyde **10 **on radicle growth of *Sorghum bicolor* at 5 x 10^-4^ mol L^-1^.

As can be observed in Table 2, radicle promotion was absent at 5 x 10^-4^ mol L^-1^, and in this case maximal inhibition (Figure 2) was achieved with the α,β-unsaturated aldehyde **10 **(52% inhibition). Importantly, in our previous investigation [21] the α,β-unsaturated aldehyde **4 **(Figure 1) displayed the highest inhibitory activity against *Sorghum bicolor* at 1 x 10^-3^ mol L^-1^. 

Although not statistically significant, hydrogenation of the double of compound **10** resulted in decreasing inhibitory activity. Overall, these results emphasized the importance of the formyl group for enhancing phyototoxic activity against the monocotyledonous species investigated herein. Compared to *Cucumis sativus*, the observed phyototoxic activity for compound **6** was depressed, and removal of the double bond significantly enhanced the inhibitory activity of this ester. 

Lastly, the reduction of the carbonyl functionality to the more polar hydroxyl group did not improve the desired phytotoxic activity. For instance, whilst compound **6** inhibited the radicle growth of *Sorghum bicolor* by 13.33%, the hydroxylated derivative **17** exhibited only 2% inhibition. 

## Conclusions

In summary, several stable oxabicycles were prepared using the [4+3] cycloaddition reaction and functional group interconversions. The synthesized compounds displayed phytotoxic effects on both monocotyledonous and dicotyledonous species, with various patterns of biological action. The most active compounds possessed a common structural motif: the presence of a carbonyl group conjugated conjugated with a double bond. At the concentration tested, the most active compound exhibited 52% of inhibition against the monocotyledonous species *Sorghum bicolor*. 

## Experimental

### General

All reactions were carried out under a protective atmosphere of dry nitrogen. Reagents and solvents were purified, when necessary, according to procedures described by Perrin and Armarego [31]. The compound 2,4-dibromopentan-3-one (**5**) was obtained from the bromination of the commercially available pentan-3-one (Aldrich - Milwaukee, WI) according to procedures described in the literature [17]. The preparation of 3-hydroxymethyl-2-methylfuran was carried out as previously reported [22]. Pyridinium chlorochromate (PCC) was purchased from Aldrich, and utilized without further purification. The ^1^H- and ^13^C-NMR spectra were recorded on a Varian Mercury 300 instrument (300 MHz and 75 MHz respectively), using deuterated chloroform as a solvent and tetramethylsilane (TMS) as internal standard (*δ* = 0). IR spectra were obtained using a Perkin Elmer Paragon 1000 FTIR spectrophotometer, using potassium bromide (1% v/v) disks, scanning from 600 to 4000 cm^-1^. Mass spectra were recorded on a Shimadzu GCMS-QP5050A instrument under electron impact (70 eV) conditions. Melting points are uncorrected and were obtained from MQAPF-301 melting point apparatus (Microquimica, Brazil). Analytical thin layer chromatography analysis was conducted on aluminum backed precoated silica gel plates. Column chromatography was performed over silica gel (60-230 mesh).

### Synthesis of 7-(methoxycarbonyl)-1,2α,4α-trimethyl-8-oxabicyclo[3.2.1]oct-6-en-3-one *(**6**)*

To a two neck round-bottomed flask (100 mL), fitted with a 10 mL dropping funnel, were added dry acetonitrile (20 mL) and sodium iodide (4.92 g, 32.8 mmol) with vigorous stirring under a slow stream of nitrogen. The mixture was cooled to 0 °C and powdered copper (1.56 g, 24.6 mmol) was added, followed by methyl 5-methylfuran-2-carboxylate (1.5 g, 10.66 mmol). A solution of 2,4-dibromo-pentan-3-one (**5**) (2.0 g, 8.2 mmol) dissolved in dry acetonitrile (30 mL) was added, *via* a dropping funnel, over 1h at 0 °C. The reaction mixture was allowed to warm up to rt. The mixture was stirring for 18h and cooled down to 0 °C before addition of dichloromethane (40 mL) and water (40 mL). The mixture was extracted with dichloromethane (3 x 30 mL) and filtered through a Celite pad. The mother liquor was washed with aqueous NH_4_OH (35%, 50 mL), brine (30 mL), dried over anhydrous MgSO_4_ and concentrated under reduced pressure. The resulting material was purified by column chromatography on silica gel eluted with hexane-diethyl ether 6:1 v/v. This procedure afforded compound **9** as a colorless crystal solid in 51% yield (610 mg, 2.72 mmol). TLC: R_f_ = 0.20 (hexane-diethyl ether 7:1 v/v); mp 44.1**-**45.5 ºC; IR (cm^-1^), *V*_max_: 2981, 2940, 2876, 1722, 1714, 1614, 1438, 1379, 1343, 1265, 1227, 1165, 1121, 1090, 1056, 1025, 978, 916, 862, 762, 694; ^1^H-NMR: *δ* 0.98 (d, 3H, *J* = 7.2 Hz, H-10), 1.05 (d, 3H, *J* = 6.9 Hz, H-9), 1.69 (s, 3H, 1-CH_3_), 2.66 (q, 1H, *J* = 6.9 Hz, H2), 2.85 ( dq, 1H, *J* = 5.1 Hz and *J* = 7.2 Hz, H4), 3.71 (s, 3H, CH_3_OCO-), 4.87 (dd, 1H, *J* = 2.1 Hz and *J* = 5.1 Hz, H5), 7.12(d, 1H, *J* = 2.1 Hz, H6); ^13^C**-**NMR: *δ* 9.77 (C9), 10.38 (C10), 21.34 (1-CH_3_), 49.50 (C4), 52.00 (CH_3_OCO-), 56.80 (C2), 80.94 (C5), 85.50 (C1), 141.20 (C7), 144.82 (C6), 163.24 (CH_3_OCO-), 208.58 (C3); MS, *m/z* (%): 224, C_12_H_16_O_4_, [M^+▪^], (10), 209 (7), 192 (6), 168 (27), 153 (76), 149 (13), 135 (24), 121 (18), 109 (25), 93 (10), 79 (22), 55 (25), 43 (100), 39 (44). The syntheses of oxabicycles **7-9** were accomplished using a procedure similar to that described for compound **6**, and yields obtained are presented in Scheme 1. The structural characterization of compounds **8** and **9 **has been previously described [17, 32]. The structure of *7-hydroxymethyl-1,2α,4α-trimethyl-8-oxabicyclo[3.2.1]oct-6-en-3-one* (**7**) is supported by the following spectroscopic data: Colorless solid, purified by column chromatography (eluent: hexane-diethyl ether 1:1 v/v); TLC: R_f_ = 0.32 (hexane-diethyl ether 1:1 v/v); mp 68.0–68.7 ºC; IR (cm^-1^) *V*_max_: 3600-3100 (broad band), 2978, 2937, 2875, 1708, 1629 1449, 1377, 1198, 1168, 1037, 940, 907; ^1^H-NMR: *δ* 0.90 (d, 3H, *J* = 7.2 Hz**, **H10), 1.10 (d, 3H, *J* = 7.2 Hz, H9), 1.48 (s, 3H, 1-CH_3_), 1.77 (brs, 1H, -CH_2_OH), 2.66 (q, 1H, *J* = 7.2 Hz, H2), 2.80 (dq, 1H, *J* = 4.8 Hz and *J* = 7.2 Hz, H4), 4.24–4.18 (m, 2H, -CH_2_OH), 4.80–4.74 (m, 1H, H5), 6.14–6.10 (m, 1H, H6); ^13^C**-**NMR: *δ* 9.44 (C9), 10.21 (C10), 20.57 (1-CH_3_), 49.39 (C4), 56.30 (C2), 59.72 (-CH_2_OH), 81.19 (C5), 88.00 (C1), 126.85 (C6), 150.47 (C7), 209.29 (C3); MS, *m/z* (%): 196, C_11_H_16_O_3, _[M^+▪^], (22), 181 (15), 165 (9), 139 (42), 125 (49), 122 (33), 109 (100), 95 (18), 79 (22), 55 (47), 53 (23).

### Oxidation of compound ***7*** to 7-(formyl)-1,2α,4α-trimethyl-8-oxabicyclo[3.2.1]oct-6-en-3-one *(**10**)*

A round bottom flask (10 mL) was charged with PCC (0.17 g, 0.77 mmol) and anhydrous dichloromethane (1.5 mL). The mixture was vigorously stirred and compound **7** (0.1 g, 0.51 mmol), dissolved in anhydrous dichloromethane (1.5 mL), was added. After 90 minutes, compound **7** was completely consumed (TLC analysis). Upon completion of the reaction, diethyl ether (3 mL) was added, and the black solid formed was washed with two portions of diethyl ether (5 x 2 mL). The combined organic layers were filtered, washed with a solution of sodium bicarbonate 5% (10 mL), dried over anhydrous MgSO_4_, filtered, and finally concentrated under reduced pressure. The resulting material was re-crystallized and compound **10 **was obtained as white crystals in 78% yield (77 mg, 0.4 mmol). TLC: R_f_ = 0.55 (hexane-diethyl ether 1:1 v/v); mp 75.2–76.7 ºC; IR (cm^-1^) *V*_max_: 3073, 2980, 2938, 2873, 1710, 1684, 1602, 1448, 1379, 1337, 1253, 1196, 1115, 1091, 1028, 915, 860, 708, 656, 617,530; ^1^H-NMR: *δ* 1.00 (d, 3H, *J* = 6.9 Hz, H10), 1.01 (d, 3H, *J* = 7.2 Hz, H9), 1.68 (s, 3H, 1-CH_3_), 2.67 (q, 1H, *J* = 7.2 Hz, H2), 2.91 (dq, 1H, *J* = 5.1 Hz and *J* = 6.9 Hz, H4), 4.98 (dd, 1H, *J* = 2.1 Hz and *J* = 5.1 Hz, H5), 7.15 (d, 1H, *J* = 2.1 Hz, H6), 9.76 (s, 1H, -CHO); ^13^C**-**NMR: *δ* 9.49 (C9), 10.40 (C10), 20.78 (1-CH_3_), 49.59 (C4), 56.56 (C2), 81.46 (C5), 88.43 (C1), 149.85 (C7), 152.30 (C6), 186.81 (CHO), 208.09 (C3); MS, *m/z* (%): 194, C_11_H_17_O_3,_ [M^+▪^], (12), 179 (13), 166 (19), 151 (15), 138 (76), 123 (77), 109 (100), 95 (83), 84 (86), 69 (21), 55 (71).

### Oxidation of compound ***10*** to 2,4,5-trimethyl-3-oxo-8-oxabicyclo[3.2.1]oct-6-ene-6-carboxylic acid (**11**)

In a round bottom flask were sequentially added silver oxide (0.24 g, 1.03 mmol), 10 % m/v sodium hydroxide solution (0.52 mL) and water (1 mL). The mixture was kept at room temperature, and under continuous stirring. Subsequently, compound **10** was added in one portion (0.10 g, 0.51 mmol) and the resulting reaction mixture was stirred for 90 minutes followed by filtration. The solid residue was washed with hot water followed by dichloromethane. The phases were separated, and to the aqueous layer the filtrate, concentrated hydrochloric acid was added until pH = 3. The aqueous layer was extracted with dichloromethane (4 x 10 mL) and the organic extracts were combined. The resulting organic layer was dried over anhydrous MgSO_4_, filtrated and concentrated under reduced pressure. After purification of the residue by silica gel column chromatography (hexane-diethyl ether 1:3 v/v) compound **11** was obtained as a white solid in 29% yield (0.031 g; 0.15 mmol). TLC: Rf = 0.30 (hexane-diethyl ether 1:3 v/v); mp 139.4–141 ºC; IR (cm^-1^) *V*_max_: 3600-2500 (Broad band), 1702, 1677, 1602, 1571, 1458 1378, 1303, 1267, 1202, 1097, 1026 917, 862, 807, 777, 691, 514; ^1^H-NMR: δ 1.00 (d, 3H, J = 7.2 Hz, H10), 1.08 (d, 3H, J = 7.2 Hz, H9), 1.70 (s, 3H, 1-CH_3_), 2.68 (q, 1H, J = 7.2 Hz, H2), 2.88 (dq, 1H, J = 5.1 Hz and J = 7.2 Hz, H4), 4.91 (dd, 1H, J = 2.1 Hz and J = 5.1 Hz, H5), 7.29 (d,1H, J = 2.1 Hz, H6); ^13^C**-**NMR: δ 9.84 (C9), 10.41 (C10), 21.28 (1-CH_3_), 49.48 (C4), 56.8 (C2), 81.01 (C5), 88.39 (C1), 140.88 (C7), 147.86 (C6), 167.23 (-COOH), 208.43 (C3); MS, m/z (%): 210, [M^+▪^], C_11_H_14_O_4_, (18), 195 (9), 192 (3), 177 (17), 165 (3), 153 (44), 139 (100), 135 (20), 109 (67), 55 (68).

### Hydrogenation of compound ***6*** to 7-(methoxycarbonyl)-1,2α,4α-trimethyl-8-oxabicyclo[3.2.1]oct-6-en-3-one *(**12**)*

A solution of **6** (300 mg; 1.34 mmol) and ethyl acetate (34 mL) was placed in a round bottom flask, under hydrogen atmosphere, along with 10 % Pd/C (62 mg). The reaction mixture was stirred at 40 ^o^C for 22h. After this time the catalyst was filtered off through a Celite pad and the solvent removed under reduced pressure. The reduced product **12** was obtained as a pale yellow oil in 92% yield (0.28 g; 1.24 mmol) after purification by silica gel column chromatography (hexane-diethyl ether 7:1 v/v). TLC: R_f_ = 0.30 (hexane-ethyl acetate 7:1 v/v); IR (cm^-1^) *V*_max_: 2976, 2949, 2876, 1739, 1717, 1437, 1379, 1354, 1266, 1206, 1163, 1123, 1099, 1066, 1041, 1011, 994, 951, 927, 860, 760, 517; ^1^H-NMR: *δ* 0.87 (d, 3H, *J* = 7.2 Hz, H9), 1.01 (d, 3H, *J* = 6.9 Hz, H10), 1.61 (s, 3H, 1-CH_3_), 221-2.15 (m, 2H, H6), 2.58 (q, 1H, *J* = 7.2 Hz, H2), 2.86-2.74 (m, 2H, H4 and H7), 3.64 (s, 3H, CH_3_OCO-), 4.40 (br dd_, _1H, *J* = 4.8 Hz and *J* = 9.6 Hz, H5); ^13^C**-**NMR: 8.88 (C9), 10.00 (C10), 25.78 (1-CH_3_), 29.72 (C6), 50.03 (C4), 52.00 (CH_3_OCO-), 52.60 (C7), 54.87 (C2), 79.27 (C5), 87.08 (C1), 171.34 (CH_3_OCO-), 208.03 (C3); MS, *m/z* (%): 226, [M^+▪^], C_12_H_18_O_4_, (10), 195 (8), 142 (5), 127 (79), 123 (5), 111 (18), 95 (38), 81 (9), 57 (21), 43 (100). The compounds 7-hydroxymethyl-1,*2α,4α*-trimethyl-8-oxabicylo[3.2.1]-octan-3-one (**13**) and 7-formyl-1,*2α,4α-*trimethyl-8-oxabicyclo[3.2.1]octan-3-one (**14**) were synthesized using a similar procedure to that described for **12** and yields are shown in Scheme 2. The structures of **13** and **14** are supported by the following spectroscopic data: *7-hydroxymethyl-1,2α,4α-trimethyl-8-oxabicyclo[3.2.1]oct-6-an-3-one* (**13**). White solid, purified by silica gel column chromatography eluted with hexane-ethyl acetate 1:2 v/v; TLC: R_f_ = 0.29 (hexane-ethyl acetate 1:2 v/v); mp 118.3–119.7 ºC IR (cm^-1^) *V*_max_: 3462, 2970, 2956, 2932, 2894, 1706, 1458, 1402, 1372, 1334, 1256, 1187, 1091, 1068, 1039, 1020, 953, 922, 881, 802, 658, 527; ^1^H-NMR: *δ* 0.96 (d, 3H, *J* = 6.9 Hz, H10), 1.07 (d, 3H, *J* = 7.2 Hz, H9), 1.49 (s, 3H, 1-CH_3_), 1.74 (s, 1H, -CH_2_OH), 2.25-2.27 (m, 3H, H6 and H7), 2.57 (br s, 1H, H2), 2.73 (br s_, _1H, H4), 3.59 (d, 2H, *J* = 6 Hz, -CH_2_OH), 4.34 (br s, 1H, H5); ^13^C**-**NMR: 8.84 (C9), 9.65 (C10), 25.48 (1-CH_3_), 31.32 (C6), 50.49 (C4), 52.05 (C7), 55.17 (C2), 62.17 (-CH_2_OH), 79.08 (C5), 86.91 (C1), 209.92 (C3); MS, *m/z* (%): 198, [M^+▪^], C_11_H_18_O_3_, (6), 180 (1), 168 (2), 125 (5), 111 (17), 95 (7), 81 (23), 67 (13), 55 (26), 43 (100); *7-formyl-1,2α,4α-trimethyl-8-oxabicyclo[3.2.1]oct-6-an-3-one* (**14**). White solid, purified by silica gel column chromatography eluted with hexane-ethyl acetate 2:1 v/v; TLC: R_f_ = 0.25 (hexane-ethyl acetate , 2:1 v/v); mp 59.3–60.1 ºC; IR (cm^-1^) *V*_max_: 2977, 2943, 2877, 2736, 1715, 1459, 1381, 1320, 1282, 1164, 1124, 1072, 1032, 1018, 991, 880, 779, 706, 518; ^1^H- NMR: *δ* 0.97 (d, 3H, *J*** = **6.9 Hz, H9), 1.03 (d, 3H, *J* = 7.2 Hz, H10), 1.61 (s, 3H, 1-CH_3_), 2.10-2.00 (m, 1H, H6a), 2.24-2.14 (m, 1H, H6b),2.70 (q, 1H, *J* = 6.9 Hz, H2), 2.84-2.76 (m, 1H, H4), 2.96-2.88 (m, 1H, H7), 4.56-4.50 (m, 1H, H5), 9.6 (s, 1H, CHO); ^13^C**-**NMR: *δ* 9.45 (C9), 9.87 (C10), 25.57 (1-CH_3_), 27.56 (C6), 50.50 (C4), 55.09 (C2), 62.39 (C7), 80.36 (C-5), 89.27 (C1), 199.02 (-CHO), 209.28 (C3); MS, *m/z* (%): 196, [M^+▪^], C_11_H_17_O_3_, (3), 139 (2), 125 (2), 111 (18), 97 (32), 86 (27), 72 (12), 56 (29), 43 (100), 32 (33).

### Reduction of compound ***7*** to 7-hydroxymethyl-1,2α,4α-trimethyl-8-oxabicyclo[3.2.1]oct-6-en-3α-ol *(**15**)*

In a two neck round bottom flask were added the compound **7** (0.15 g; 0.77 mmol), NaBH_4_ (45 mg; 2.29 mmol) and ethanol (10 mL). The mixture was maintained under nitrogen atmosphere and reflux at 55 ^o^C for 5h when water (20 mL) was added. The aqueous layer was extracted with dichloromethane (3 x 20 mL). The organic extracts were combined and the resulting organic layer was dried over anhydrous MgSO_4_, filtered, and concentrated under reduced pressure. After purification by silica gel column chromatography (hexane-ethyl acetate 1:10 v/v) followed by re-crystallization, compound **15** was obtained as a white solid in 83% yield (0.19 g; 0.6 mmol). TLC: R_f_ = 0.32 (hexane-ethyl acetate , 1:10 v/v); mp 125.3–126.2 ºC; IR (cm^-1^) *V*_max_: 3100-3650 (Broad band), 2965, 2930, 2905, 2877, 1636, 1453, 1377, 1346, 1247, 1201, 1160, 1143, 1129, 1084, 1041, 1012, 964, 935, 912, 861, 851, 800, 723, 528; ^1^H-NMR: *δ* 0.94 (d, 3H, *J* = 7.5 Hz, H9), 1.05 (d, 3H, *J* = 7.5 Hz, H10), 1.28 (s, 3H, 1-CH_3_), 2.03 (dq, 1H, *J* = 4.8 Hz and *J* = 7.5 Hz, H2), 2.25-2.15 (m, 1H, H4), 3.72 (t, 1H, *J* = 4.8 Hz, H3), 4.11 (br d, 1H, *J* = 14.2 Hz, -CH_2_OH), 4.28 (br d, 1H, *J* = 14.2 Hz, -CH_2_OH), 4.42 (br s, 1H, H5), 6.26 (br d, 1H, *J* = 1.5 Hz, H6).; ^13^C-NMR: *δ* 12.87 (C9), 13.01 (C10), 20.63 (1-CH_3_), 38.92 (C4), 44.09 (C2), 58.99 (-CH_2_OH), 73.58 (C3), 81.81 (C5), 85.86 (C1), 130.51 (C6), 150.75 (C7); MS, *m/z* (%): 198 [M^+▪^], C_11_H_18_O_3_, (4), 180 (1), 165 (4), 139 (32), 125 (23), 110 (32), 95 (13), 83 (10), 69 (12), 55 (14), 43 (100). The procedure described above was also applied to the reduction of compounds **6 **and **9** and the yields are presented in Scheme 2. In the case of compound **6**, a complex mixture was obtained from which compound **17** was isolated. The structure of compound **16 **and **17** is supported by the following spectroscopic data: *1,2α,4α,5-tetramethyl-8-oxabicyclo[3.2.1]oct-6-en-3α-ol* (**16**). Pale yellow oil purified by silica gel column chromatography eluted with hexane-diethyl ether 1:1 v/v; TLC: R_f_ = 0.30 (hexane-diethyl ether, 1:1 v/v); IR (CsI, cm^-1^) *V*_max_: 3561, 2967, 2930, 2872, 1455, 1441, 1407, 1371, 1264, 1214, 1165, 1139, 1040, 1021, 965, 844, 764, 737, 690, 619, 527; ^1^H- NMR: *δ* 1.00 (d, 6H, *J* = 6.9 Hz, H9/H10), 1.36 (s, 6H, 1-CH_3­_/5-CH_3_), 2.00 (dq, 2H, *J* = 4.3 Hz and *J* = 6.9 Hz, H2/H4), 3.6 (t, 1H, *J*** = **4.3 Hz, H3), 6.2 (s, 2H, H6/H7); ^13^C-NMR: *δ* 13.45 (C9/C10), 22.12 (1-CH_3_/5-CH_3_), 44.41 (C2/C4), 74.24 (C3), 87.14 (C1/C5), 139.27 (C6/C7); MS, *m/z* (%):182 [M^+▪^], C_11_H_18_O_2_, (1), 167 (2), 149 (6), 124 (15), 109 (16), 95 (7), 84 (100), 51 (58); *methyl 3-hydroxy-2,4,5-trimethyl-8-oxabicyclo[3.2.1] oct-6-en-3α-ol-carboxylate* (**17**). White solid, purified by column chromatography eluted with hexane-diethyl ether 1:1 v/v; TLC: R_f_ = 0.33 (hexane-diethyl ether 1:1 v/v); mp 108–109. ºC; IR (cm^-1^) *V*_max_: 3488, 2970_, _2934, 2880, 1741, 1619, 1454, 1380, 1357, 1335, 1308, 1276, 1234, 1196, 1161, 1084, 1031, 989, 967, 895, 768, 706, 678, 551, 526; ^1^H-NMR: *δ* 0.97 (d, 3H, *J* = 7.2 Hz, H10), 1.06 (d, 3H, *J* = 7.2 Hz, H9), 1.20 (s, 3H, 1-CH_3_), 1.82 (dq, 1H, *J* = 4.2 Hz and *J* = 7.2 Hz, H2), 2.061-2.11 (m, 1H, H4), 2.28-2.32 (m, 2H, H6), 3.48 (dd, 1H, *J* = 6.0 Hz and *J* = 6.3 Hz, H7), 3.68 (s, 3H, -OCOCH_3_), 3.70 (dt, 1H, *J* = 1.0 Hz and *J* = 4.2 Hz, H3), 4.17-4.11 (m, 1H, H5); ^13^C-NMR: *δ* 12.83 (C9), 12.97 (C10), 21.28 (1-CH_3_), 30.51 (C6), 38.88 (C4), 45.34 (-OCOCH_3_), 46.34 (C2), 51.47 (C7), 72.30 (C3), 78.50 (C5), 84.07 (C1), 175.44 (-OCOCH_3_); MS, *m/z* (%):228 [M^+▪^], C_12_H_20_O_4_, (1), 210 (6), 197 (2), 179 (4), 159 (40), 127 (100), 111 (22), 95 (54), 69 (15), 55 (60).

### Reduction of compound ***14*** to 7-hydroxymethyl-1,2α,4α-trimethyl-8-oxabicyclo[3.2.1]octan-3α-ol *(**18**)*

A two necked round bottom flask in ice bath and under nitrogen atmosphere was charged with LiAlH_4_ (43 mg; 1.14 mmol) and anhydrous THF (3 mL). Subsequently, a solution of compound **14** (150 mg; 0.75 mmol) in anhydrous THF (2 mL) was added dropwise to the LiAlH_4_ suspension in THF. Stirring was maintained for 3.5 h after which water (2 mL) followed by 15% m/v NaOH aqueous solution (2 mL) and more water (10 mL) were added. The granular solid that was formed was filtered off. The organic layer was extracted with dichloromethane (3 x 10 mL) and the extracts were combined. The resulting organic layer was dried over anhydrous MgSO_4_, filtered, and concentrated under reduced pressure. The resulting residue was purified by silica gel column chromatography (hexane-ethyl acetate 1:7 v/v) and the compound **18** was obtained as a yellow solid in 75% yield (110 mg, 0.56 mmol). TLC: R_f_ = 0.26 (hexane-ethyl acetate 1:7 v/v); mp 119.2–120.4 ºC; IR (cm^-1^) *V*_max_: 3600–3100 (Broad band), 2965, 2931, 2885, 1459, 1379, 1323, 1287, 1197, 1167, 1130, 1083, 1010, 972, 892, 799, 690, 524; ^1^H-NMR: *δ* 0.95 (d, 3H, *J* = 7.5 Hz, H9) 1.03 (d, 3H, *J* = 7.5 Hz, H10), 1.33 (s, 3H, 1-CH_3_), 2.25-1.93 (m, 5H, H6, H7, H2 and H4), 3.66 (s, 2H, OH), 3.73 (dt, 1H, *J* = 1.2 Hz and *J* = 4.5 Hz, H3), 3.88 (dd, 1H, *J* = 5.4 Hz and *J* = 11.3 Hz, -CH_2_OH), 3.96 (dd, 1H, *J* = 4.2 Hz and *J* = 11.3 Hz, -CH_2_OH), 4.05–4.0 (m, 1H, H5); ^13^C-NMR: *δ* 12.77 (C10), 12.81 (C9), 25.77 (1-CH_3_), 27.00 (C6), 39.90 (C2), 45.30 (C4), 51.05 (C7), 59.78 (-CH_2_OH), 72.85 (C3), 77.74 (C5), 81.31 (C1); MS, *m/z* (%):182 (1), 131 (3), 113 (41), 95 (7), 81 (23), 67 (13), 55 (26), 43 (100). For this compound, the molecular ion peak was not observed.

### Plant Growth Inhibition Assays

Stock solutions were prepared for the bioassays by dissolving a proper amount of each compound in xylene (48 mL) and pentan-3-one (16 mL). After the addition of the surfactant Tween 60 (1 drop), the resulting suspension was transferred to a volumetric flask and water-diluted, so as to obtain a final concentration of 5 x 10^-4^ mol L^­-1^. A mixture with the same composition as described above, but without the compound to be evaluated, was used as control. Groups of 6 pre-germinated *Sorghum bicolor* L. or *Cucumis sativus* L. seeds were placed in Petri Dishes (i.d. = 9 cm) with acid washed sand (150 g) and the solution (20 mL) containing the compound to be tested. The Petri dishes were sealed with Parafilm® and incubated at 28 ^o^C, in the dark and placed at 75^o^ angle in relation to the surface of the incubator. After 48 hours, the root length was measured to the nearest millimeter. All treatments were replicated four times in a completely randomized design. The percentage of root growth inhibition was calculated in relation to the root length of the control. The date was analyzed using Tukey’s test at 0.05 probability level [33].
